# Epoxide Hydrolases: Multipotential Biocatalysts

**DOI:** 10.3390/ijms24087334

**Published:** 2023-04-15

**Authors:** Marek Bučko, Katarína Kaniaková, Helena Hronská, Peter Gemeiner, Michal Rosenberg

**Affiliations:** 1 Department of Glycobiotechnology, Institute of Chemistry, Center for Glycomics, Slovak Academy of Sciences, Dúbravská cesta 9, 845 38 Bratislava, Slovakia; peter.gemeiner@savba.sk; 2 Institute of Biotechnology, Faculty of Chemical and Food Technology, Slovak University of Technology, Radlinského 9, 812 37 Bratislava, Slovakia; katarina.kaniakova@stuba.sk (K.K.); helena.hronska@stuba.sk (H.H.); michal.rosenberg@stuba.sk (M.R.)

**Keywords:** epoxide hydrolase, epoxides, chiral precursors, biocatalysis, enantioselectivity, enzyme engineering, stabilization, immobilization, enzyme cascade

## Abstract

Epoxide hydrolases are attractive and industrially important biocatalysts. They can catalyze the enantioselective hydrolysis of epoxides to the corresponding diols as chiral building blocks for bioactive compounds and drugs. In this review article, we discuss the state of the art and development potential of epoxide hydrolases as biocatalysts based on the most recent approaches and techniques. The review covers new approaches to discover epoxide hydrolases using genome mining and enzyme metagenomics, as well as improving enzyme activity, enantioselectivity, enantioconvergence, and thermostability by directed evolution and a rational design. Further improvements in operational and storage stabilization, reusability, pH stabilization, and thermal stabilization by immobilization techniques are discussed in this study. New possibilities for expanding the synthetic capabilities of epoxide hydrolases by their involvement in non-natural enzyme cascade reactions are described.

## 1. Introduction

Epoxide hydrolases (EHs) catalyze the hydrolysis of the oxirane ring by adding a water molecule to form the corresponding vicinal diol without requiring any cofactor [1]. Most EHs were members of the broad superfamily of hydrolases with an α/β-fold with the assigned EC number 3.3.2.3 in the BRENDA database. However, the classification was deleted from this database, and EHs were divided into two new enzyme groups: EC 3.3.2.9, microsomal EHs, and EC 3.3.2.10, soluble EHs. There are also EHs (e.g., limonene-1,2-hydrolase or leukotriene-A4 hydrolase) with completely different structures and catalytic mechanisms that are classified separately [2].

Epoxide hydrolases are found in various organisms, from prokaryotes to eukaryotes. They perform different functions depending on their site of localization and the origin of the organisms. Among eukaryotes, mammalian EHs are the most studied, mainly because of their role in xenobiotic metabolism and signaling processes [3,4]. Prokaryotic EHs are necessary for catabolic pathways in which specific aromatic compounds or alkenes are used as carbon sources [5].

Due to their broad substrate specificity and high stereospecificity, epoxide hydrolases have received attention as industrial biocatalysts. The EH-catalyzed enantioselective hydrolysis of epoxides was first discovered in mammalian cells, but the application of EHs from this source on an industrial scale was limited due to their low availability. Large quantities of EHs could be prepared after the discovery of microbial EH producers and their recombinant expression in host organisms. This opened the way to their commercial use in the industrial production of chiral compounds, especially in the synthesis of enantiopure drugs, chiral epoxides, and diols [6]. The enantioselectivity and stability of microbial EHs can also be improved by using organic solvents, detergents, ionic liquids, immobilization, and by innovative methods, such as enzyme engineering or direct evolution methods [7].

Other reviews published on EHs have focused on their sources, substrate scope, enantioselectivity, and application in organic chemistry [1,8,9,10]. In this review, we summarized and updated the most interesting applications of EHs associated with the formation of enantiopure epoxides or diols from chiral precursors, which are key intermediates for synthesizing various target products. We also presented the latest approaches for modifying these enzymes by enzyme engineering techniques used to produce tailored EHs. We provided examples of successful applications of immobilized EHs and the benefits of immobilization methods. Finally, we highlighted the recent successful involvement of EHs in enzymatic cascade reactions, confirming the multipotential use of EHs.

## 2. Epoxides and Diols as Chiral Precursors and Their Applications

Chirality is an important property of bioactive molecules, especially in pharmacology, where different stereoisomers can have different pharmacological properties. Many epoxides and diols are intermediates in the synthesis of many drugs, although epoxides are often produced as racemic mixtures. Approximately 57% of commercially available drugs and approximately 99% of purified natural products are chiral compounds [11]. Chiral chemicals are in high demand commercially, and the global market for chiral drugs is expected to grow in the future [12].

Chiral epoxides can serve as useful intermediates for synthesizing many chemicals with industrial applications. Optically pure epoxides can be prepared via biocatalysis using two major approaches. The first approach includes the direct epoxidation of alkenes and vicinal halohydrins using monooxygenases, chloroperoxidases, and haloalcohol dehalogenases. The second method involves the hydrolysis of racemic epoxides using EHs by kinetic resolution or enantioselective hydrolysis. Among the enzymes mentioned above, the use of enantioselective EHs for producing chiral epoxides has several advantages over other enzymes. The main advantages are that EHs do not require cofactors or additional nucleophiles for their function, they are ubiquitous in nature, and they can be easily cloned and produced in large quantities [13]. The first industrial application of EHs was described in 1969 for the production of L-tartaric acid and *meso*-tartaric acid using whole bacterial cells [6]. The microbial production of L-(+)-tartaric acid was successfully commercialized in the late 1990s, and microbial methods are now considered to be more economical for producing both optical isomers of this organic acid from *cis*-epoxysuccinic acid [14]. In the 1990s, new EHs capable of enantioselective and enantioconvergent hydrolysis of structurally diverse epoxides were also discovered, which attracted the interest of organic chemists [15,16,17].

*Trans*-vicinal diols, products of EH-catalyzed reactions, also have many interesting synthetic applications. Some of the chiral precursors that can be prepared using EHs and the products that can be synthesized from them are presented in Table 1.

## 3. Natural and Recombinant EHs

Epoxide hydrolases (EHs) are ubiquitous in nature, but concerning their application in the industry, microbial enzymes are better suited for mass production. Therefore, novel EHs are mainly searched for among microorganisms. Since the discovery of the first enantioselective microbial EH, many new EH-producing organisms have been identified [6]. In the 1990s and 2000s, many EHs were discovered by the enrichment screening of isolates [27,46,47] or screening strains from various collections [22,48,49,50]. Through extensive screening, EH activity in bacteria was found to be associated with the genera *Rhodococcus*, *Nocardia*, *Mycobacterium*, and *Arthrobacter* [51]. Although the screening of microbial isolates is very laborious, it is still widely performed to discover new EHs [52,53,54].

The classic screening method has many limitations. One of them is the screening for enzyme activity, where the reaction substrates and products are identified by GC or HPLC after the samples are extracted from culture or reaction media [48]. To overcome this problem, different spectrophotometric methods for rapid activity assays were developed and used to easily determine the substrate or product. Some of these assays are the 4-(p-nitrobenzyl) pyridine assay (blue assay) [55], adrenaline assay (red assay) [56], and sodium metaperiodate assay [57].

Besides traditional culture-based methods used for screening microorganisms for enzyme activity, two new approaches for discovering novel enzymes emerged: genome mining and metagenomics.

One of the new methods is genome mining. Advancements in genome sequencing, bioinformatics, and the large number of genome sequences deposited in public databases enabled the discovery of uncharacterized biocatalysts [58]. About one-fifth of the total microbial genome in the databases is predicted to contain one or more putative EHs. Van Loo et al. [59] also showed that genome databases could be used as a source of novel EHs [59]. Stojanovski et al. [60] identified 29 putative EHs from the genomic data of six soil bacteria using genome mining. Eight of them were recombinantly expressed in *E. coli* and used for activity studies, where five were identified as α/β-fold EHs, and three showed sequence similarity to the rare class of limonene epoxide hydrolases (LEHs) [60].

Another new approach used for searching for EHs is metagenomics, i.e., the direct extraction and cloning of DNA from natural environments without culturing isolated microorganisms [61]. This method is not only used to identify novel putative EHs [62] but also to recombinantly express these genes, characterize enzymes and use them for enantioselective and regioselective hydrolysis [63,64]. The metagenomic approach can also be useful for discovering enzymes with extremophilic properties. Two novel LEHs and two α/β-fold EHs from environmental DNA were obtained from hot spring environments. Although all DNA samples were collected at around neutral pH and at lower temperatures (from 46 to 65 °C) compared to other hot spring samples, all four novel EHs had higher thermal stability than any other EHs and LEHs isolated from natural environmental sources [65,66].

Advancements in genetic engineering allowed the recombinant expression of EHs in various hosts to produce larger quantities of EHs and for easier purification. Different types of expression vectors, including mammalian and insect cell lines, have been used to express EHs. However, EHs are generally expressed in microbial cells, most often in *Escherichia coli* [10]. Along with the discovery of novel EHs and advances in recombinant techniques, various techniques have been implemented to enhance their properties through enzyme engineering, immobilization, and optimization of the reaction medium.

## 4. Improvement of EHs by Enzyme Engineering

One of the main constraints in the application of enzymes in the industry is their insufficient enantioselectivity, narrow substrate specificity, low activity, and thermal stability [67]. This problem could be overcome by using enzyme engineering techniques. Enzyme engineering refers to the process of modifying the amino acid sequences of enzymes to change their properties, such as catalytic activity, thermostability, organic solvent tolerance, and substrate and reaction specificity [68].

The relatively good knowledge of amino acid sequences, reaction mechanisms, and structures of various EHs [69,70,71,72] allowed the application of various methods of enzyme engineering of EHs. After the initial application of directed evolution to prepare stereoselective lipase [73], Cedrone et al. [74] were the first to achieve EH engineering, where they performed error-prone PCR to prepare mutants of *A. niger* EH (AnEH) with a 3.3-fold increase in the catalytic efficiency toward 4-(*p*-nitrophenoxy)-1,2-epoxybutane [74]. Since then, many researchers have used various methods of enzyme engineering to obtain EH mutants with enhanced properties. Some of these methods include error-prone PCR [75] saturation mutagenesis [76], DNA shuffling [77], iterative saturation mutagenesis (ISM) [78,79,80], computational design [81,82], and machine learning [83].

Enhancing enzyme activity and broadening their substrate spectrum are the main objectives of enzyme engineering. The modification of selected amino acid (AA) residues in the substrate-binding pocket of EH from *Bacillus megaterium* ECU 1001 enhanced activity toward bulky racemic epoxides by 6 to 430 times. It helped perform the bioresolution of various racemic epoxides to prepare (*S*)-epoxides, which are the precursors to various β-blockers, on a preparative scale [84].

The enantioselectivity of EHs has attracted the interest of most researchers in these biocatalysts, but enzymes often lack sufficient enantioselectivity or the ability to perform enantioconvergent hydrolysis. Hence, many studies focused on increasing the enantioselectivity or improving the enantioconvergence of EHs using enzyme engineering methods (Table 2).

Along with studies on the improvement of enzyme activity, enantioselectivity, and enantioconvergence, studies on enzyme engineering have been conducted to produce EHs with higher thermostability [83,108,109]. Gumulya et al. [109] used several rounds of ISM to generate mutants with higher thermal robustness. Mutation sites in AnEH were selected based on the B-FIT approach, where the criterion for selecting AA residues was the highest B factors available from the X-ray crystallography data. The best variant showed a 21 °C increase in T^60^_50_, the temperature at which 50% of enzyme activity is lost following heat treatment for 60 min, which represents an 80-fold improvement in enzyme half-life at 60 °C [109].

A computational design was also used for generating thermostable mutants of EH. In this case, the Framework for Rapid Enzyme Stabilization by Computational Libraries (FRESCO) was applied. It is a promising computationally guided approach for protein thermostabilization that uses the Rosettaddg, FoldX, and Disulfide Discovery software packages. The apparent melting temperature of the two best multisite mutants of LEH from *R. erythropolis* with 10–12 point mutations increased from 50 °C to ~85 °C [108]. Eight AA residues, which were expected to be sensitive to changes in thermostability based on the previous study [108], and eight AA residues that were expected to be sensitive to changes in enantioselectivity selected in the TCSM ISM study [80] were selected for ISM to prepare LEH mutants with enhanced thermostability, activity, and opposite enantioselectivity against cyclohexene oxide [94].

From the perspective of industrial application, the enhancement of thermostability of *cis*-epoxysuccinate hydrolase (CESH) is also interesting. Using a semi-rational design, combining directed evolution, simulated mutagenesis, and saturation mutagenesis, the half-life of the best CESH mutant at 50 °C increased from 8.5 min to 293.2 min, and T^15^_50_ increased from 44 °C to 64.8 °C. Additionally, the effective working range of the pH of the mutant extended to 5.0–10.0 from 8.0–9.0 for the wild-type enzyme [110].

Enzyme engineering can also be applied to completely change the catalytic activity of the enzyme. Jochens et al. [111] converted the enzymatic activity within the α/β-fold hydrolase family. They reported the conversion of esterase to EH by site-directed mutagenesis, although the EH activity of modified esterase was 800-fold lower than that of the template EH from *Agrobacterium radiobacter* AD1 [111].

## 5. Immobilization of EHs

According to IUPAC, immobilization in biotechnology is the technique used for the physical or chemical fixation of cells, organelles, enzymes, or other proteins on a solid support, in a solid matrix, or retained by a membrane to increase their stability and facilitate their repeated or continued use [112]. Immobilization techniques belong to the main pillars of optimization procedures for biotransformations. The application of these techniques for EHs includes the most common immobilization principles, such as (1) adsorption, (2) covalent bonding, (3) crosslinking, (4) entrapment, and (5) encapsulation. Immobilization is also considered to be an important step in the commercialization of EHs as it provides reusability and commercial value to the process [7]. Even the first commercial preparation with EH activity was an immobilized enzyme preparation derived from *Rhodococcus* sp. called SP 409, developed by NOVO Industry. The product was originally designed as a biocatalyst for the hydrolysis of nitriles. The EH activity was later discovered by Hechtberger et al. [113], and it was used for the asymmetric hydrolysis of various racemic epoxides [113]. The commercial availability of EHs is limited, although EHs from *Rhodococcus rhodochrous* and *Aspergillus niger* are commercially available as lyophilized powder [114].

The analysis of the processes to immobilize EHs [7] showed that while adsorption and entrapment techniques were used for immobilizing whole cells with EHs (see also [115,116,117]), covalent bonding and cross-linking were used for EHs as isolated or partially purified enzymes. An exception is the encapsulation technique, which is universally suitable for stabilizing *Nocardia tartaricans* cells [116,118] using CESH and EH isolated from the *Sphinogomonas* strain [119].

As shown in Table 3, most of the immobilized epoxide hydrolases are used as purified or partially purified enzymes, immobilized on different supports by the covalent binding method. Due to the stability of EHs and their cofactor independence, the application of purified enzymes is advantageous. One exception is the low stability of CESH, which is therefore used as a whole-cell biocatalyst for enantioselective production of L-(+)-tartrate. To overcome the problems of low cell permeability, various surface displaying systems for the mentioned CESH have been developed [120]. In some cases, whole-cell immobilization is occasionally performed, where cells are immobilized by entrapment and encapsulation within porous or semipermeable microparticles with high water content.

The list of developed immobilization techniques presented in Table 3 indicates that immobilization for EHs is desirable, and its application is increasing. Several innovative methods and new materials for immobilizing EHs have been developed in the past five years. For example, CESH was immobilized by metal-ion affinity interaction with Ni-IDA agarose particles to improve enzyme thermostability and pH stability [134]. Additionally, commercial immobilization matrices were used for the covalent binding of epoxide hydrolase from *Vigna radiata* [19]. The thermostability and operational stability of the EH immobilized later during the production of β-blocker Nifenalol was improved. Some researchers have also synthesized amino-modified mesocellular silica activated by glutaraldehyde [131]. The later immobilization method enabled the improvement of operational stability and thermostability of EH from red mung beans and cutinase from *Fusarium* sp. ICT SAC1 during enantioselective and regioselective model biotransformations. The development of organic–inorganic hybrid epoxide hydrolase nanoflowers represents a promising novel immobilization concept [144]. The EH nanoflowers impart unique properties, such as a high EH concentration, an optimum conformation for EH, low mass transfer limitations, and a high surface area, which can increase enzyme activity and stability.

The advantages of immobilizing EHs, presented in Table 3, explain why immobilization techniques are still in high demand. Additionally, immobilization techniques might also significantly enhance the biocatalytic efficiency of EHs as a part of enzyme cascades, mentioned in the next chapter. Though the results achieved by enzyme cascades with EHs are limited, the immobilization of recombinant *E. coli* cells with an overproduced cascade of the enzyme halohydrin dehalogenase and epoxide hydrolase using an adsorption technique yielded promising results [139].

**Figure 1:**
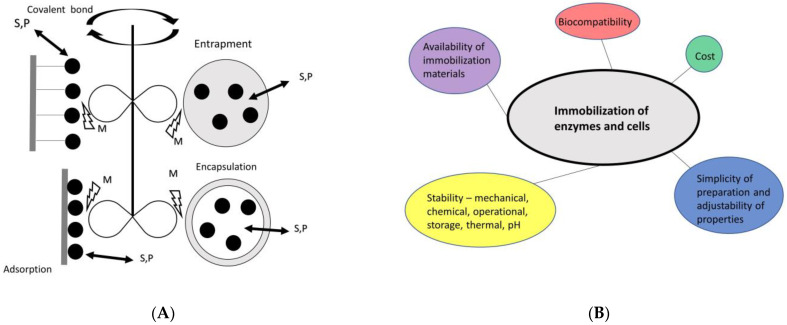
(**A**) Differences between immobilization techniques based on covalent bonding and adsorption (left) or entrapment and encapsulation (right) from the perspective of applied mechanical forces (M) in the bioreactor represented by a rotating impeller. Mechanical abrasion was applied directly on the adsorbed or covalently bond biocatalyst (left). Entrapment and encapsulation matrices protected biocatalysts from direct mechanical forces (right). The contact of immobilized biocatalysts with substrates (S) and products (P) was direct, using covalent bonding and adsorption as immobilization principles. Entrapment and encapsulation require diffusion of S and P through the immobilization matrix or the semipermeable membrane. (**B**) The five most important aspects for choosing the immobilization technique for biocatalysts.

There are also variations in immobilization protocols and the level of their characterization. Directly comparing the biocatalytic efficiency and other properties of different immobilized preparations of EHs is challenging. The latter might be the reason why the choice of immobilization technique for EHs is difficult. There are no general recommendations for the use of immobilization techniques. Selecting a proper immobilization system requires the individual consideration of several parameters, including the type of applied mechanical forces in bioreactors (Figure 1A). The general properties of immobilization systems, which are frequently evaluated and considered to be important for the successful utilization of immobilized biocatalysts, are schematically represented in Figure 1B.

## 6. Whole-Cell Cascade Biotransformations Using Microbial Epoxide Hydrolases

**Figure 2:**
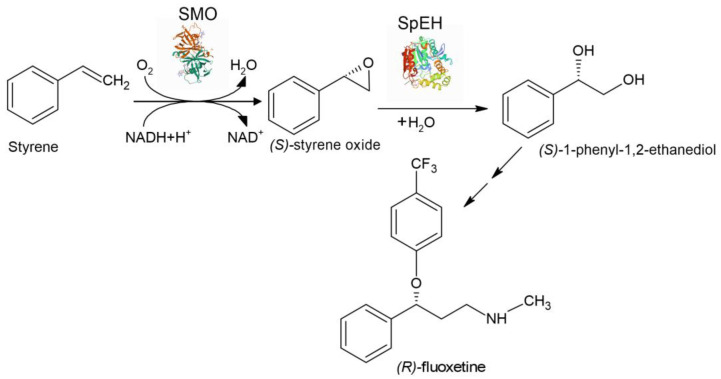
An example of a non-natural enzyme cascade consisting of two steps, including styrene monooxygenase (SMO) in the form of resting *E. coli* cells and epoxide hydrolase (SpEH) as a cell-free extract for the production of (*S*)-1-phenyl-1,2,ethanediol [152] as an intermediate for synthesizing the pharmaceutical (*R*)-fluoxetine [153].

Intensive research on the use of epoxide hydrolases (EHs) from microbial sources started in 1991 [149]. The advantage of EHs is that they do not require cofactors for the enantioselective hydrolysis of epoxides to the desired vicinal diols and enantiomerically pure epoxides. Hence, they can be used as fresh whole native cells, lyophilizates, and recombinant cells with overproduced EHs. Thus, the regeneration of cofactors and isolation of enzymes are not required; these processes are expensive and can reduce their stability [149]. The main benefit of using EHs is their ability to catalyze enantioconvergent reactions, which allow the economically efficient production of the desired substances [7]. Besides the hydrolysis of epoxides to vicinal diols, racemic mixtures of epoxides can be resolved into pure enantiomers. The synthetic possibilities of EHs were extended by the discovery of the acceptance of non-natural nucleophiles instead of water molecules in epoxide hydrolysis catalyzed by EHs, leading to the aminolysis and azidolysis of epoxides to form the corresponding amino- and azido- derivatives [150]. The need for enantioconvergent approaches for producing pure stereoisomers of vicinal diols from racemates of epoxides led to the application of the advantages of modern enzyme catalysis techniques, which resulted in the construction of artificial enzyme cascades [151]. The process involved EHs in a non-natural enzyme cascade reaction with at least one other enzyme, which facilitated the one-pot production of diols, amino alcohols, and other specialty chemicals. The importance of the two-step enzyme cascade for the biocatalytic production of chiral vicinal diol [152] as an intermediate for the chemoenzymatic synthesis of pharmaceutical (*R*)-fluoxetine for treating psychiatric and metabolic disorders is shown in Figure 2 [153].

By involving EHs in whole-cell cascade systems, the general advantages of enzyme cascades over single-step biotransformations can be used, which are as follows: (1) reaction intermediates do not have to be isolated, which makes the process cheaper and helps in performing reactions with unstable intermediates; (2) higher product yield; (3) saving resources; (4) reduction of waste production; (5) avoiding the use of toxic compounds, which are consumed immediately in situ; (6) solutions for possible enzyme inhibition issues. Additionally, the use of EH-triggered enzyme cascades can expand the catalytic capabilities of EHs, which are indicated by spontaneous cyclizations associated with the formation of new C-O bonds catalyzed by EHs [154]. In recent years, the application of EHs as biocatalysts for epoxide hydrolysis in cascade reactions has shown several possibilities and advantages, for example, reversing the enantioselectivity of the reaction by properly designing the enzyme cascade. The applications of EHs are summarized in Table 4.

**Figure 3:**
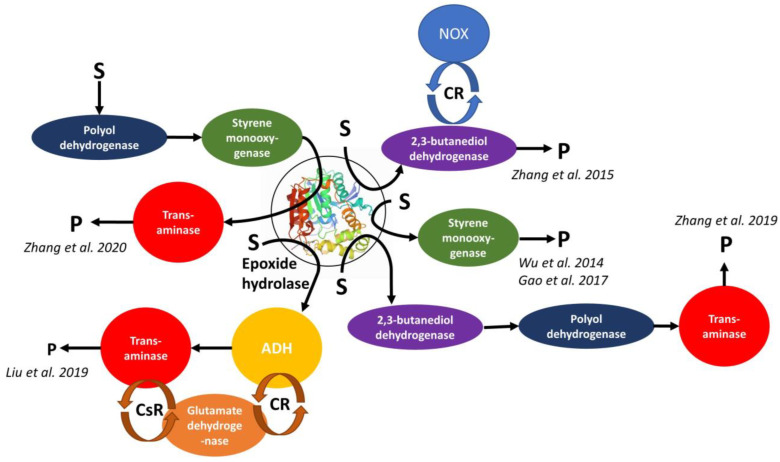
The importance and schematic incorporation of epoxide hydrolase SpEH from *Sphingomonas* sp. HXN-200 into five independent, non-natural enzyme cascades consisting of two, three, and four enzymes, co-expressed and used in the form of five whole-cell systems, as mentioned in Table 4; S: substrate, P: product, CR: cofactor regeneration, CsR: co-substrate regeneration, NOX: NADH oxidase, and ADH: alcohol dehydrogenase [155,156,158,160,161,162].

As shown in Table 4, microbial EHs represent a powerful biocatalytic tool, the synthetic possibilities of which can be further expanded by involvement in cascades. The usefulness of EHs can be increased, for example, by incorporating concrete epoxide hydrolase from *Sphingomonas* sp. HXN-200 (SpEH) into five differently designed enzyme cascades shown in Figure 3 and listed in Table 4. In this review article [163], other recent studies on new cascades using a tandem of EHs with styrene monooxygenases (SMO) connected with other enzymes to form cascades were presented. This confirmed the multifunctionality of EHs for the production of important building blocks and other chemical specialties using enzyme cascades. An important step in further applying the potential of EHs involves the development of optimization procedures leading to industrial processes.

## 7. Conclusions

The research and development of epoxide hydrolases have advanced significantly, especially their application in biocatalysis. The trend involves the expansion of the biocatalytic repertoire of epoxide hydrolases by involving them in enzyme cascades. The development of new immobilization techniques led to the improvement of the functional properties of epoxide hydrolases, especially concerning their stabilization. Classical screening techniques for new epoxide hydrolases have been replaced by more efficient approaches, especially enzyme metagenomics. Similarly, the techniques for controlling the enantioselectivity and thermostability of epoxide hydrolases are characterized by a higher degree of specificity. The development of these techniques involves the transition from directed evolution to a semi-rational design and a rational design. The progress in the field of epoxide hydrolase development is a prerequisite for its more intensive use in the industrial production of chiral building blocks, which in turn can be used for synthesizing important drugs.

## Figures and Tables

**Table 1:** Chiral precursors that can be prepared by EHs and the products that can be synthesized from them.

Chiral Precursor	Final Product	Application of Final Product	Reaction Comment	Ref.
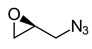	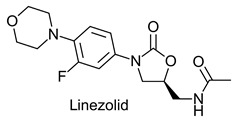	Synthetic oxazolidinone antibiotic effective against Gram positive bacteria	Selective hydrolysis of (*S*)-enantiomer	[18]
	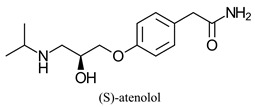	Cardio selective β-blocker for treatment of high blood pressure and heart associated chest pain		
	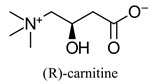	Dietary supplement, involved in long-chain fatty acid transport in cells		
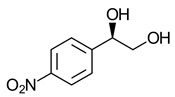	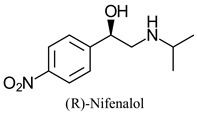	β-adrenergic blocker with antianginal and antiarrhythmic properties.	Chemo-enzymatic enantioconvergent synthesis	[19,20]
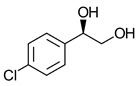	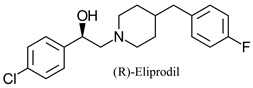	Neuroprotective agent (aspartate receptor antagonist)	Sequential bi-enzymatic hydrolysis using 2 enantiocomplementary EHs	[21]
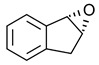	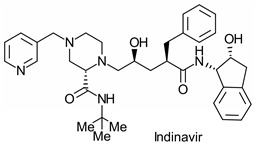	HIV protease inhibitor MK 639	Selective hydrolysis of 1(*R*),2(*S*)-enantiomer	[22]
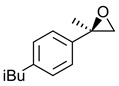	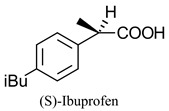	Non-steroidal anti-inflammatory drug	Selective hydrolysis of (*R*)-enantiomer	[23]
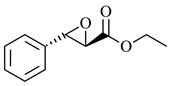	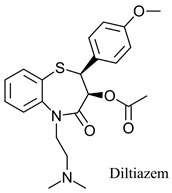	Calcium channel blocker	Kinetic resolution	[24]
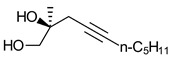	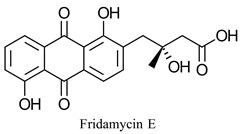	Anthracycline antibiotic with chemotherapeutic properties	Enzymatic deracemization for production of (*S*)-diol used for chemical synthesis	[25]
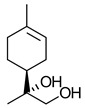	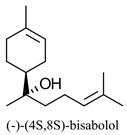	Major constituent of *Matricaria chamomilla* essential oil; ingredient for skin creams, lotions, ointments with anti-inflammatory, bactericidal and antimycotic properties	Chemo-enzymatic process for production of all 4 stereoisomers of bisabolol	[15]
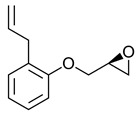	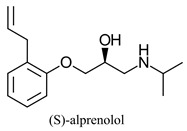	β-adrenergic receptor blocking drugs	Selective hydrolysis of (*R*)-enantiomer	[26]
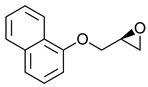	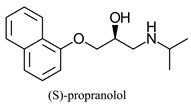			
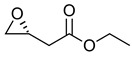	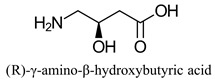	Neuromediator with antiepileptic and antihypertensive activities	Selective hydrolysis of (*S*)-enantiomer	[27]
	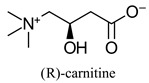	Dietary supplement, involved in long-chain fatty acid transport in cells		
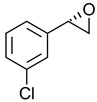	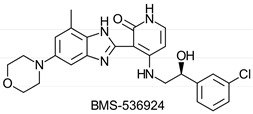	IGF-1R kinase inhibitor	Selective hydrolysis of (*R*)-enantiomer	[28]
	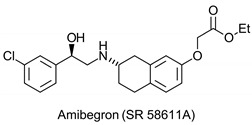	β_3_-adrenergic receptor agonists	Enantioconvergent hydrolysis of racemic epoxide	[29]
	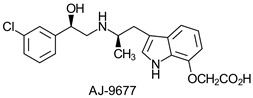			
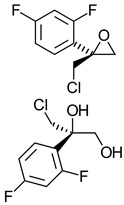	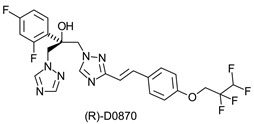	Antifungal triazole drug	Production of optically pure epoxide and diol that can be used for chemical synthesis of optically pure triazole derivatives	[30,31]
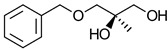	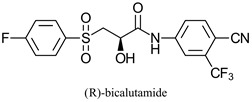	Non-steroidal antiandrogen drug used for treatment of prostate cancer	Chemo-enzymatic synthesis of optically pure diol	[32]
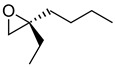	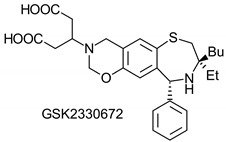	Ileal bile acid transport (iBAT) inhibitor indicated for diabetes type II	Kinetic resolution	[33]
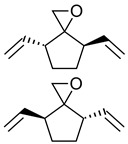	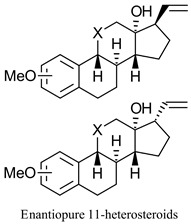	Chiral precursors for synthesis of various steroidal compounds	Kinetic resolution to produce both enantiomers of spiroepoxide, using 2 different EHs	[34]
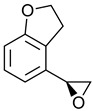	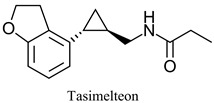	Melatonin receptor agonist used for treatment of sleep disorders	Selective hydrolysis of (*R*)-enantiomer	[35,36]
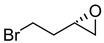	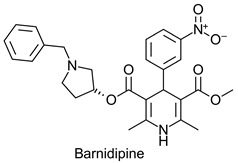	Calcium channel blocker used for treatment of hypertension	Selective hydrolysis of (*R*)-enantiomer	[37]
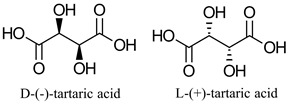		Chiral chemical building block with broad applications in chemical, pharmaceutical, food industries	Asymmetric hydrolysis to produce optically pure diol	[14]
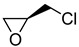	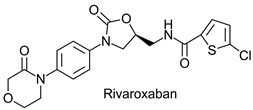	Anticoagulant; direct factor Xa inhibitor developed by Bayer and marketed as Xarelto	- ^1^	[38,39]
	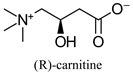	Dietary supplement, involved in long-chain fatty acid transport in cells	-	[40,41]
	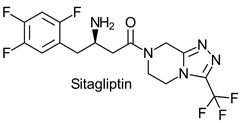	Antidiabetic drug	-	[42]
	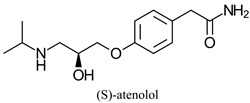	β-adrenergic receptor blocking drug	-	[43]
	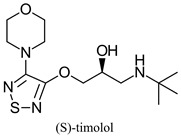	β-adrenergic antagonist drug	-	[44,45]

^1^ Only potential application.

**Table 2:** The summary of EHs engineered for higher enantioselectivity and improved enantioconvergence.

Modified Property	Source of EH Used as Template for Mutagenesis	Enzyme Engineering Method	Mutant	Substrate	E (-)		*ee*_P_ (%)		C (%)		Ref.
					Mutant	WT	Mutant	WT	Mutant	WT	
Enantioselectivity	*Agrobacterium radiobacter* AD1	Directed evolution—Error-prone PCR and DNA shuffling	F108I/P205H/Y215H/E271V	styrene oxide	>50	16	NI ^1^		NI		[85]
				*p*-nitrostyrene oxide	81	56	NI		NI		
				*p*-nitrophenyl glycidyl ether	32	3.4	NI		NI		
				epichlorohydrin	40	<2	NI		NI		
				1,2-epoxyhexane	27	3.6	NI		NI		
		Directed evolution—DNA shuffling and site-saturation mutagenesis	I219F	styrene oxide	91 (*R*)	17 (*R*)	NI		NI		[77]
		Rational design—Site-saturation mutagenesis	F108I	*p*-nitrophenyl glycidyl ether	20 (*S*)	3.4 (*S*)	NI		NI		[86]
			F108T		22 (*S*)	3.4 (*S*)	NI		NI		
	*Agromyces mediolanus* ZJB120203	Rational design—Structure-based site saturation and site-directed mutagenesis	W182F/S207V/N240D	epichlorohydrin	90.0 (*R*)	12.9 (*R*)	NI		NI		[87]
	*Aspergillus niger* LCP 521	Directed evolution—one round of error-prone PCR	A217V/K332E/A390E	phenyl glycidyl ether	10.8 (*S*)	4.6 (*S*)	74 (*S*)	56 (*S*)	39	33	[75]
		Semi-rational design —Directed evolution using ISM with combinatorial active site saturation (CASTing)	L215F/A217N/R219S/L249Y/T317W/T318V/M329P/L330Y/C350V	phenyl glycidyl ether	115 (*S*)	4.6 (*S*)	95 (*S*)	56 (*S*)	48	33	[88]
		Semi-rational design—Directed evolution using ISM and optimalization of expression in recombinant cells	P221S/F244C/L249F/L215F/T317F/T318V(ProThrAlaSerAlaProHisThrTyrArgGluPheIle)-L349V ^2^	phenyl glycidyl ether	160 (*S*)	4.6 (*S*)	97 (*S*)	56 (*S*)	45	33	[89]
		Semi-rational design—Directed evolution using all 24 possible pathways using 4 randomization sites for ISM	L215F/R219V/L249F/T317F/T318C/L349D/C350Y	phenyl glycidyl ether	158 (*S*)	4 (*S*)	98 (*S*)	61 (*S*)	30	28	[90]
	*Aspergillus usamii* (AuEH2)	Semi-rational design—Microtuning of the substrate-binding pocket	A214C/A250I	styrene oxide	202	16	>99 (*R*)	NI	50.2	40	[91]
			A250I	*o*-nitrostyrene oxide	341	96	98.0 (*R*)	NI	50.5	NI	
			A250W	isopropyl glycidyl ether	204	6.8	80.2 (*S*)	NI	55.2	NI	
	*Rhodococcus erythropolis* DCL14	Directed evolution—ISM using NDT codon degeneracy	M32C/I80F/L114C/I116V	cyclopentene oxide	NI		93 (*S*,*S*)	13 (*R,R*)	65	72	[92]
				cyclohexene oxide	NI		97 (*S*,*S*)	4 (*S*,*S*)	94	84	
				cycloheptene oxide	NI		98 (*S*,*S*)	17 (*S*,*S*)	75	74	
				*cis*-2,3-butene oxide	NI		93 (*R*,*R*)	5 (*S*,*S*)	NI		
				phenyl glycidyl ether	32 (*R*)	2.6 (*R*)	92 (*R*)	37 (*R*)	31	33	
				styrene oxide	44 (*S*)	2.8 (*R*)	91 (*S*)	40 (*R*)	43	36	
			M32C/L74I/M78F/I80C/V83I	cyclopentene oxide	NI		80 (*R*,*R*)	13 (*R,R*)	81	72	
				cyclohexene oxide	NI		90 (*R*,*R*)	4 (*S*,*S*)	74	84	
				cycloheptene oxide	NI		77 (*R*,*R*)	17 (*S*,*S*)	84	74	
				*cis*-2,3-butene oxide	NI		83 (*R*,*R*)	5 (*S*,*S*)	NI		
				styrene oxide	36 (*R*)	2.8 (*R*)	91 (*R*)	40 (*R*)	30	36	
		Directed evolution—ISM using a single-code saturation mutagenesis (SCSM)	L74F/M78F/L103V/L114V/I116V/F139V/L147V	cyclohexene oxide	NI		92 (*S*,*S*)	4(*S*,*S*)	>99	84	[78]
				cycloheptene oxide	NI		94 (*S*,*S*)	17 (*S*,*S*)	52	97	
			L74F/M78F/I80V/L114F	cyclohexene oxide	NI		96 (*R*,*R*)	4 (*S*,*S*)	83	84	
				cycloheptene oxide	NI		94 (*R*,*R*)	17 (*S*,*S*)	66	97	
		Directed evolution—ISM using double-code saturation mutagenesis (DCSM)	L74F/M78F/I80F/L114V/I116V/F138V	cyclopentene oxide	NI		85 (*S*,*S*)	13 (*R*,*R*)	13	84	[79]
				cyclohexene oxide	NI		97 (*S*,*S*)	4 (*S*,*S*)	98	>99	
				cycloheptene oxide	NI		97 (*S*,*S*)	17 (*S*,*S*)	73	97	
			M78V/I80V/L114F	cyclohexene oxide	NI		92 (*R*,*R*)	13 (*R*,*R*)	99	>99	
				cycloheptene oxide	NI		85 (*R*,*R*)	4 (*S*,*S*)	40	97	
				styrene oxide	NI		57 (*S*)	21 (*R*)	7	46	
		Directed evolution—ISM using triple-code saturation mutagenesis (TCSM)	I80Y/L114V/I116V	cyclohexene oxide	NI		99 (*S*,*S*)	4 (*S*,*S*)	97	>99	[80]
				cycloheptene oxide	NI		98 (*S*,*S*)	17 (*S*,*S*)	81	97	
				styrene oxide	28.0	1.8	92 (*S*)	26 (*R*)	15	17	
			M32V/M78V/I80V/L114F	cyclohexene oxide	NI		97 (*R*,*R*)	4 (*S*,*S*)	>99	>99	
				cycloheptene oxide	NI		94 (*R*,*R*)	17 (*S*,*S*)	83	97	
		Semi-rational design—Directed evolution using ISM with reduced AA alphabets using binary pattern based on choosing hydrophobic and hydrophilic amino acids	I80F/V83I/L114 V/I116V	cyclopentene oxide	NI		94 (*S*,*S*)	7 (*R,R*)	34	69	[93]
				cyclohexene oxide	NI		97 (*S*,*S*)	3 (*S*,*S*)	93	87	
				cycloheptene oxide	NI		97 (*S*,*S*)	22 (*S*,*S*)	96	99	
			I80V/V83I/L114 V	cyclopentene oxide	NI		51 (*R*,*R*)	7 (*R*,*R*)	48	69	
				cyclohexene oxide	NI		79 (*R*,*R*)	3 (*S*,*S*)	97	87	
				cycloheptene oxide	NI		53 (*R*,*R*)	22 (*S*,*S*)	99	99	
		Semi-rational design—Directed evolution using ISM with the aim to improve thermostability, enantioselectivity and activity	T76K/L114V/I116V/N92K/F139V/L147F/S15D/A19K/L74F/M78F/E45D	cyclohexene oxide	NI		94 (*S*,*S*)	2 (*S*,*S*)	100	100	[94]
			S15P/M78F/N92K/F139V/T76K/T85K/E45D/I80V/E124D	cyclohexene oxide	NI		80 (*R*,*R*)	2 (*S*,*S*)	100	100	
		Semi-rational design—Directed evolution using saturation mutagenesis, mutants were prepared by high-fidelity solid-phase chemical gene synthesis on silicon chips followed by efficient gene assembly instead of PCR to overcome AA bias	M78F/I80Y/L114V/I116V	cyclohexene oxide	NI		>98 (*S*,*S*)	NI	>98	NI	[95]
	*R. erythropolis DCL14* (mutant LEH-P) ^3^	Rational design—Computational design of mutant library using CASCO strategy	M32L/L74I/I80V/L103F/F139L	cyclopentene oxide	NI		85.5 (*R*,*R*)	23.9 (*R*,*R*)	NI		[96]
			M32L/L35W/L74F/M78F/I80A/I116V/F139L		NI		90.2 (*S*,*S*)	23.9 (*R*,*R*)	NI		
		Rational design—Use of Rosetta enzyme design to computationally predict enantioselective mutants and high-throughput-multiple independent molecular docking simulations for in silico screening of the generated mutant libraries	M32A/M78I/I80F/L103I/I116V/F139L	cyclopentene oxide	NI		85 (*S*,*S*)	14 (*R*,*R*)	NI		[81]
			L35W/L74F/I80G/I116V/F139L	*cis*-2,3-butane oxide	NI		82 (*S*,*S*)	2 (*S*,*S*)	NI		
			M32L/L35G/I80W/L103V/F139L	*cis*-stilbene oxide	NI		>99 (*R*,*R*)	92 (*R*,*R*)	98	NI	
			M32L/L35M/L103I/L114M/I116F/F139L		NI		88 (*S*,*S*)	92 (*R*,*R*)	63	NI	
	*Solanum tuberosum* (StEH1)	Semi-rational design—Directed evolution—ISM targeting AA residues around active site of enzyme	W106L/L109Y/V141K/I155V	(2,3-epoxypropyl)benzene	15 (*R*)	0.4 (*R*)	60 (*R*)	32 (*S*)	NI		[97]
		Semi-rational design—Directed evolution with 2 rounds of iterative saturation mutagenesis	W106L/L109Y/V141K/I155W/F189C	styrene oxide	5800 (*S*)	69 (*S*)	NI		NI		[98]
				*trans*-2-methylstyrene oxide	770 (*S*)	84 (*S*)	NI		NI		
	*Sphingomonas* sp. HXN-200	Semi-rational design—Site-directed mutagenesis of selected AA residues in active site based on homology modelling	V196A/N226A/M332A	phenyl glycidyl ether	21.2 (*R*)	2.2 (*R*)	79.2 (*S*)	61.9 (*S*)	50	50	[99]
	metagenomic DNA (Kau2EH)	Semi-rational design—Directed evolution by randomizing selected sites within substrate binding pocket	V290Y	*p*-chlorostyrene oxide	130	NI	97 (*R*)	NI	50	NI	[100]
Enantioconvergence	*A. niger* M200	Semi-rational design—ISM, mutated sites were chosen on structural similarity with EH from *A. niger* LCP 521	L349V/C350W/T317W/T318V/M218W/R219E/L215M/A217G/M245A	styrene oxide	22	10	70.1 (*R*)	3.0 (*R*)	100	100	[101]
				*p*-chlorostyrene oxide	20	40	70.5 (*R*)	4.4 (*R*)	100	100	
	*Glycine max* (GmEH3)	Semi-rational design —Site-saturation and combinatorial mutagenesis used for reshaping substrate-binding pocket	W102V/P187F	1,2-epoxyhexane	NI		83.8 (*R*)	47.2 (*R*)	>99	>99	[102]
	*Phaseolus vulgaris* (PvEH1)	Rational design—Site-directed mutagenesis based on molecular docking simulations and multiple alignment	L105I/M160A/M175I	styrene oxide	3.6	1.5	87.8 (*R*)	33.6 (*R*)	NI		[103]
				m-chlorostyrene oxide	NI		69.7 (*R*)	1.0 (*R*)	NI		
				*p*-nitrostyrene oxide	NI		64.7 (*R*)	50.3 (*R*)	NI		
				*m*-nitrostyrene oxide	NI		52.3 (*R*)	14.7 (*R*)	NI		
				*p*-chlorostyrene oxide	NI		70.9 (*R*)	51.4 (*R*)	NI		
		Rational design—Leucine scanning used for identification of AA residues at sites lining the enzyme’s binding pocket responsible for enantioconvergence and subsequent saturation mutagenesis	L105I/M160A/M175I/Y149L/P184W	*m*-chlorostyrene oxide	NI		96.1 (*R*)	1.0 (*R*)	>99	>99	[104]
		Rational design—Reshaping of substrate binding pocket	L105I/V106I/M160A/M175I/S178T/P184W	styrene oxide	NI		90.3 (*R*,*R*)	33.6 (*R*,*R*)	>99.9	99.1	[82]
				*p*-nitrostyrene oxide	NI		86.7 (*R*,*R*)	50.3 (*R*,*R*)	84.2	99.3	
				*m*-nitrostyrene oxide	NI		85.1 (*R*,*R*)	14.7 (*R,R*)	>99.9	99.7	
				*p*-fluorostyrene oxide	NI		90.6 (*R*,*R*)	13.6 (*R*,*R*)	>99.9	98.7	
				*m*-chlorostyrene oxide	6	2	96.2 (*R*,*R*)	1.0 (*R*,*R*)	99.2	99.9	
	*Rhodotorula paludigena* JNU001	Rational design—Microtuning substrate-binding pocket of EH by computer-aided design using valine scanning mutagenesis	L360C	*m*-nitrostyrene oxide	NI		93.4 (*R*)	85.7 (*R*)	99	>99	[105]
	*Vigna radiata* (VrEH2)	Rational design—Creation of smart library by site-directed mutagenesis using reduced AA alphabet to prepare enantioconvergent EH	M263N	*p*-nitrostyrene oxide	NI		98 (*R*)	84 (*R*)	99.5	NI	[106]
				*m*-nitrostyrene oxide	NI		90 (*R*)	20 (*R*)	>99	>99	
		Rational design—Creation of smart library by site-directed mutagenesis using reduced AA alphabet to prepare enantioconvergent EH	M263Q	*m*-chlorostyrene oxide	NI		90 (*R*)	20 (*R*)	NI		[107]
			M263V	2-naphthyloxirane	NI		90	60	NI		
	metagenomic DNA (Kau2EH)	Semi-rational design —Directed evolution by randomizing selected sites within substrate binding pocket	W110L/F113L/F161Y/P193G/V290W	*p*-chlorostyrene oxide	17	23	93 (*R*)	84 (*R*)	100	100	[100]

^1^ No information. ^2^ Amino acids in parentheses were randomly inserted into the protein sequence during error-prone PCR. ^3^ Thermostable mutant LEH-P of LEH from *Rhodococcus erythropolis* DCL14 [108] was used as the template for mutagenesis.

**Table 3:** Immobilization techniques, materials, and their benefits for EHs.

Immobilization Technique	EH (Source)	Immobilized Biocatalyst	Support	Benefit of Immobilization	Ref.
Covalent bond	ArEH (*Agrobacterium radiobacter* AD1)	Crude enzyme extract	LX-1000EP modified by EDA LX-1000EP	Operational stability, reusability, increased thermal stability as compared to free enzyme	[121]
		Purified enzyme	Dextran activated with NaIO_4_ and ethylene glycol Ficoll activated with NaIO_4_ and ethylene glycol Amylopectin activated with NaIO_4_ and ethylene glycol Carboxymethyl cellulose activated with NaIO_4_ and ethylene glycol	Improved tolerance to the inhibitory effects of Co^2+^, Fe^3+^ and EDTA	[122]
	Kau2EH (metagenomic DNA)	Purified enzyme	Eupergit C 250L Eupergit C Eupergit C modified by IDA and CuSO_4_ Sepabeads EC-EP Sepabeads EC-EP modified by IDA and CuSO_4_	Significantly higher thermal stability as compared to free enzyme	[123]
	VrEH2_M263N_ (*Vigna radiata*)	Purified enzyme	ECR8205F (Epoxy) ECR4204F (Epoxy) ECR8215F (Epoxy) ES-103 (Epoxy) ESR-1 (Amino) ESQ-1 (Amino) ECR8405F (Amino)	Improvement of thermal and operational stability as compared to free enzyme	[19]
	AnEH (*Aspergillus niger* LCP 521)	Purified enzyme (lyophilized powder)	Eupergit C Eupergit C 250L Eupergit C 250L modified by EDA Eupergit C modified by IDA and CuSO_4_	Improvement of enzyme stability and enantioselectivity	[124]
			Eupergit C 250L modified by EDA and glutaraldehyde	Improvement of enzyme storage and thermal stability and enantioselectivity	[125]
			Eupergit C modified by EDA and glutaraldehyde; Florisil^®^ silanized with 3-APTES and activated with glutaraldehyde	Improvement of enzyme reusability and enantioselectivity	[126]
			Epoxide-derived silica gel	Enhancement of enzyme stability in the presence of DMSO	[127]
	StEH (*Solanum tuberosum*)	Crude enzyme extract (lyophilized powder)	Sepabeads EP—Epoxy modified by IDA and CuSO_4_ Glyoxyl–agarose (agarose modified by glycidol and oxidized by NaIO_4_)	Stabilization of enzyme	[128]
	mEH (rat liver)	Purified enzyme	Sephadex G-150 activated by 1,1′-carbonyldiimidazole	Enhancement of stability and repeated use of the enzyme	[129]
			Dextran activated by 1,1′-carbonyldiimidazole	Increasement of enzyme stability	[130]
	VaEH (*Vigna angularis*)	Partially purified enzyme	Mesocellular foam silica (MCF) amino modified and activated by glutaraldehyde; Santa Barbara Amorphous (SBA-15) amino modified and activated by glutaraldehyde	Enhancement of enzyme operational stability and thermal stability	[131]
Ionic bond/Affinity bond (His-tag)	StEH (*Solanum tuberosum*)	Crude enzyme extract	Silica oxide powder modified by resacetophenone and Co^2+^	Observation of enzyme activity in organic solvents	[132]
	mMcEH (triple mutant) (*Mugil cephalus*)	Purified enzyme	NiO presenting magnetic nanoparticles	Reusability of enzyme	[133]
	CESH (*Nocardia tartaricans* CAS-52)	Purified enzyme	Metal ion affinity chromatography media Ni-IDA QZT 6FF	Enhancement of enzyme activity	[134]
Adsorption	AnEH (*Aspergillus niger* LCP 521)	Purified enzyme	Accurel EP 100 (polypropylene resin)	Enhancement of enzyme operational stability using nonporous DEAE-cellulose	[135]
			DEAE cellulose (ionic bond)	Reusability of enzyme	[135,136]
			Porous polypropylene	Immobilized for preparative purposes (reuse, continuous reactor)	[137]
			Lewatit^®^ VP OC 1600	Enzyme reusability, enhancement of enantioselectivity	[126]
	Nsp.EH (*Nocardia* sp. EH1)	Partially purified enzyme	DEAE cellulose (ionic bond)	Enzyme stabilization	[138]
	ArEH (*Agrobacterium radiobacter* AD1 expressed in *E. coli*)	Whole cells	Perlite	Immobilized for preparative purposes	[139]
	McEH (*Mugil cephalus*)	Purified enzyme	Magnetically separable silica Mag-MSU-F (adsorption) + cross-linking with glutaraldehyde	Enhancement of enzyme stability and reusability	[140]
CLEA	VrEH (*Vigna radiata*)	Partially purified enzyme extract	Cross-linker: glutaraldehyde	Enhancement of catalytic efficiency, enantioselectivity and product yield	[141]
				Enhancement of initial reaction rate, product yield, enantioselectivity, operational stability	[142]
Co-polymerization	RgEH (*Rhodotorula glutinis* CIMW 147 (ATCC 201718))	Partially purified enzyme	Acylation of enzyme by itaconic acid, bio-imprinted with substrate and copolymerized with ethylene glycol dimethacrylate	Enzyme stabilization, reusability and product separation, improvement of enantioselectivity	[143]
Nanoflowers	GmEH (*Glycine max*)	Purified enzyme	Organic–inorganic nanoflowers formed with Ca^2+^ ions	High catalytic activity and stability	[144]
Metal–organic framework (MOFs)	GmEH (*Glycine max*)	Crude enzyme preparation (extract)	UiO-66-NH_2_ metal−organic framework (MOF) cross-linked with glutaraldehyde	Higher enzyme pH stability, thermostability and tolerance to organic solvents as compared to free enzyme	[145]
	HdEH (*Hypsibius dujardini*)	Purified enzyme	Zeolitic imidazole frameworks (ZIF-8) Zeolitic imidazole frameworks treated with glutaraldehyde (Glu/ZIF-8)	Enhancement of stability, enantioselectivity, reusability of enzyme	[146]
Encapsulation	CESH (*Nocardia tartaricans* ATCC 31191)	Whole cells	Polyelectrolyte complex microcapsules from sodium alginate−cellulose sulfate−poly(methylene-co-guanidine)	Enhanced enzyme activity, storage stability and decreased reaction time using immobilized whole cells as compared to free cells	[116]
				Enhancement of operational stability	[118]
Entrapment	RtEH (*Rhodosporidium toruloides* UOFS Y-0471)	Whole cells	Calcium alginate	Stabilization of cells	[147]
	CESH (*Labrys* sp. BK-8)	Whole cells	κ-carrageenan	Stabilization of cells	[148]
not mentioned	NOVO SP409 (*Rhodococcus* sp. commercial preparation)	Crude enzyme	Not mentioned	Preparative purposes	[113]

**Table 4:** Examples of epoxide hydrolases in the whole-cell enzyme cascades and their role in the cascades.

Enzymes in the Cascade including EH and Enzyme Source/GMO Cells	Substrate(s)	Product(s)	Note to the Role of EH in the Cascade	Ref.
Epoxide hydrolase SpEH from *Sphingomonas* sp. HXN-200 and butanediol dehydrogenase BDHA from *Bacillus subtilis* BGSC1A1 and NADH oxidase NOX from *Lactobacillus brevis* DSM 20054/ *E. coli* expressing separately SpEH and *E. coli* co-expressing BDHA-NOX *E. coli* co-expressing SpEH-BDHA-NOX	Meso- or racemic epoxides	*R*-(α)-hydroxyketones	No significant influence of using separately expressed vs. co-expressed enzymes of the cascade on *ee* and conversion	[155]
Epoxide hydrolase SpEH from *Sphingomonas* sp. HXN-200 or Epoxide hydrolase StEH from *Solanum tuberosum* and styrene monooxygenase SMO/ *E. coli* co-expressing SpEH-SMO *E. coli* co-expressing StEH-SMO	Aryl olefins	Chiral vicinal diols	The first enzyme cascade which enabled reversing enantioselectivity of dihydroxylation using StEH instead of SpEH	[156]
Epoxide hydrolase AmEH from *Agromyces mediolanus* and halohydrin dehalogenase HheC from *Agrobacterium radiobacter* AD1/ *E. coli* expressing separately HheC and AmEH *E. coli* co-expressing HheC-AmEH	1,3-dichloro-2-propanol	Chiral epichlorohydrin	Effect of co-expressed vs. separately expressed enzymes on the enantioselectivity of the cascade	[157]
Epoxide hydrolase SpEH from *Sphingomonas* sp. and styrene monooxygenase SMO from *Pseudomonas* sp./ *E. coli* co-expressing SpEH-SMO	Styrene	(*S*)-1-phenyl-1,2-ethanediol	Aqueous/organic biphasic reaction system was used for the first time for cascade biotransformation to enhance productivity	[158]
Epoxide hydrolase MupZ from *Pseudomonas fluorescens* NCIMB 10586 and Rieske non-heme oxygenase MupW *Pseudomonas fluorescens* NCIMB 10586/ *E. coli* expressing MupW *E. coli* co-expressing MupW-MupZ	Mupirocins	Hydroxylated tetrahydropyrans and tetrahydrofurans	Cascade containing epoxide hydrolase and Rieske non-heme oxygenase enabled formation of heterocyclic THP ring, which is difficult to achieve biosynthetically	[159]
Epoxide hydrolase SpEH from *Sphingomonas* sp. HXN-200, alcohol dehydrogenase MnADH from *Mycobacterium neoaurum* VKM AC-1815D, ω-transaminase PAKω-TA from *Pseudomonas aeruginosa* and glutamate dehydrogenase GluDH from *E. coli* BL21/ *E. coli* BL21 (SGMP) co-expressing 4-enzyme self-sufficient cascade system SpEH-MnADH-PAKω-TA-GluDH	(*S*)-epoxides	Chiral 1,2-aminoalcohols	The first one-step synthesis of optically pure 1,2-amino alcohols from (*S*)-epoxides employing a synthetic redox-self-sufficient enzyme cascade in recombinant cells	[160]
Epoxide hydrolase SpEH from *Sphingomonas* sp. HXN-200, 2,3-butanediol dehydrogenase BDHA from *Bacillus subtilis*, polyol dehydrogenase GoSCR from *Gluconobacter oxydans*, (R)-ω-transaminase MVTA from *Mycobacterium vanbaalenii*/ *E. coli* expressing separatelly SpEH, BDHA, GoSCR, MVTA *E. coli* co-expressing SpEH-BDHA-GoSCR-MVTA	Racemic epoxides	Enantiopure β-amino alcohols	General access to variety of chiral β-amino alcohols starting from inexpensive racemic epoxides using designed enzyme cascade process in recombinant cells	[161]
Styrene monooxygenase SMO from *Pseudomonas* sp., epoxide hydrolase SpEH from *Sphingomonas* sp. HXN-200, polyol dehydrogenase GoSCR from *Gluconobacter oxydans*, (R)-ω-transaminase MVTA from *Mycobacterium vanbaalenii* or transaminase BMTA from *Bacillus megaterium* SC6394/ *E. coli* CGS-DEM co-expressing GoSCR-SMO-SpEH-MVTA *E. coli* CGS-DEB co-expressing GoSCR-SMO-SpEH-BMTA	Styrenyl olefins	2-amino-2-phenyl ethanols	Challenging direct regio- and stereoselective aminohydroxylation of olefins to unprotected enantioenriched β-amino alcohols was enabled by novel one-pot four-enzyme biocatalytic cascade in good yields and excellent enantioselectivity	[162]

## Data Availability

Data sharing not applicable.
